# Clinical Outcomes of CDK4/6 Inhibitor Therapy in HR+/HER2− Metastatic Breast Cancer: A Multicenter Comparison of HER2-Low and HER2-Zero Subgroups

**DOI:** 10.1155/tbj/5577345

**Published:** 2025-06-27

**Authors:** Erkan Ozcan, Ivo Gokmen, Fahri Akgul, Fatma Akdag Kahvecioglu, Abdussamet Celebi, Osman Kostek, Ilhan Hacıbekiroglu, Bulent Erdogan

**Affiliations:** ^1^Department of Internal Medicine, Division of Medical Oncology, Faculty of Medicine, Trakya University, Edirne, Turkey; ^2^Department of Internal Medicine, Division of Medical Oncology, Faculty of Medicine, Sakarya University, Sakarya, Turkey; ^3^Department of Internal Medicine, Division of Medical Oncology, Faculty of Medicine, Marmara University, Istanbul, Turkey

**Keywords:** CDK4/6 inhibitors, HER2 low, HER2 zero, HR-positive breast cancer, progression-free survival

## Abstract

**Background:** The clinical impact of HER2-low status on the efficacy of cyclin-dependent kinase 4/6 inhibitor (CDK4/6i). Therapy in patients with hormone receptor-positive (HR+), HER2-negative metastatic breast cancer (MBC) remains unclear.

**Methods:** We conducted a multicenter, retrospective analysis including 212 female patients with HR+/HER2−MBC treated with CDK4/6is between 2018 and 2022. Patients were classified as HER2-zero or HER2-low based on immunohistochemistry results. Progression-free survival (PFS), objective response rate (ORR), and disease control rate (DCR) were compared between the two groups.

**Results:** Median PFS was 16.0 months in the HER2-low group and 13.9 months in the HER2-zero group (*p*=0.40). In first-line therapy, PFS was numerically longer in the HER2-low group (18.6 vs. 14.9 months; *p*=0.26) although this was not statistically significant. ORR was 71.4% in HER2-low and 62% in HER2-zero patients, and DCR was 86.6% and 82%, respectively (both *p* > 0.05). Subgroup analyses showed that within the HER2-low group, patients with ≥ 2 metastatic sites had significantly shorter PFS compared with those with a single site (14.1 vs. 20.2 months; *p*=0.02), and the presence of visceral metastases was associated with poorer PFS (*p*=0.003). Overall survival (OS) data were immature, with only 24.6% of the patients deceased at the time of analysis.

**Conclusion:** HER2 status did not significantly impact treatment outcomes with CDK4/6i in HR+/HER2-negative MBC patients. However, subgroup analyses indicated that metastatic burden, particularly the number of metastatic sites and the presence of visceral disease, may adversely influence PFS. These findings highlight the need for further validation in larger prospective studies.

## 1. Introduction

Breast cancer remains a major global health burden, representing 31% of all female cancers and 15% of cancer-related deaths [1]. Despite advances in treatment, metastatic breast cancer (MBC) continues to pose a substantial clinical challenge. The molecular heterogeneity of MBC significantly impacts prognosis and therapeutic response, necessitating more tailored approaches [2]. Among molecular subtypes, hormone receptor-positive (HR+)/HER2-negative breast cancer is the most common and is typically treated with endocrine therapy (ET) plus cyclin-dependent kinase 4/6 inhibitors (CDK4/6i). This combination has reshaped the treatment paradigm, improving progression-free survival (PFS) and overall outcomes for many patients [3].

Recent studies have identified HER2-low breast cancer as a distinct subgroup within the HER2-negative category, defined by immunohistochemistry (IHC) scores of 1+ or 2+ without HER2 gene amplification by in situ hybridization (ISH) [4]. Representing 45%–55% of HER2-negative cases [5], this subgroup has gained attention due to the efficacy of antibody-drug conjugates (ADCs), notably trastuzumab deruxtecan, in the DESTINY-Breast04 trial [6]. However, the clinical and biological significance of HER2-low status in HR+/HER2-negative MBC treated with CDK4/6i and ET remains unclear, with studies reporting either lower response rates or shorter PFS in HER2-low tumors, while others have found no significant differences compared to HER2-zero cases.

The PALOMA-2 and PALOMA-3 clinical trials demonstrated that combining CDK4/6i with ET significantly improved PFS in patients with HR+/HER2-negative MBC [7]. However, whether HER2-low patients experience similar benefits as HER2-zero patients remains unclear. Some studies report lower response rates in HER2-low tumors, while others find comparable outcomes between the two groups [8–14]. These inconsistencies highlight the need for further research into the therapeutic and prognostic significance of the HER2-low status.

HER2 expression, even at low levels, may influence the efficacy of CDK4/6i through bidirectional crosstalk with HR signaling pathways [15, 16]. This interaction can modulate HER2 protein levels independently of gene amplification, potentially altering therapeutic sensitivity or resistance. Preliminary evidence suggests that HER2-low tumors may show distinct PFS patterns compared with HER2-zero [8–14] though their prognostic significance remains unclear.

This study aims to evaluate whether HER2-low expression influences treatment response and PFS in patients with HR+/HER2-negative MBC treated with CDK4/6i and ET. We hypothesize that, due to biological interaction with HR pathways, HER2-low tumors may respond differently compared with HER2-zero tumors. By comparing these subgroups in a real-world setting, we seek to clarify this potential association and contribute meaningful insights to the current literature on treatment stratification in this heterogeneous population.

## 2. Materials and Methods

### 2.1. Study Population and Study Design

This multicenter, retrospective study evaluated clinical, pathological, and radiological data from female patients with HR+/HER2−MBC treated with CDK4/6i (palbociclib or ribociclib) between January 2018 and December 2022. Data were obtained from medical records at the Medical Oncology Departments of Trakya University, Sakarya University, and Marmara University Pendik Training and Research Hospital. The study adhered to the Declaration of Helsinki and received ethics approval from each participating center, including Trakya University Faculty of Medicine Ethics Committee (Approval no: 18/37, date: November 27, 2023).

Eligible patients were women aged ≥ 18 years with histologically or cytologically confirmed metastatic HR+/HER2−breast cancer, ER positivity ≥ 10% by IHC, and who received CDK4/6i within the first four lines of metastatic therapy. All patients underwent at least one posttreatment radiological (CT) or nuclear medicine (PET-CT, bone scintigraphy) evaluation and had complete clinical, pathological, and radiological documentation. Patients were excluded if they had incomplete data, lacked follow-up imaging, or had a follow-up period shorter than six months without disease progression or death.

### 2.2. Treatment Protocol and Monitoring

Patients received CDK4/6i combined with ET until disease progression, unacceptable toxicity, death, or patient decision. In cases of clinical benefit despite radiological progression, continuation of therapy was allowed at the treating physician's discretion, recognizing possible discrepancies between imaging findings and clinical status.

Patients were monitored from treatment initiation until death or last recorded evaluation. Disease progression was assessed using CT, PET-CT, or when necessary, magnetic resonance imaging (MRI), with imaging performed every 6–12 weeks based on clinical judgment and disease status. Response evaluation was performed according to RECIST Version 1.1 for CT and PERCIST criteria for PET-CT [17, 18]. Bone metastases, lymph node metastases, and visceral organ metastases were each considered separate metastatic sites. Follow-up concluded in December 2022, providing a sufficient observation window for evaluating treatment outcomes, including PFS, OS, and adverse events.

### 2.3. HER2 and HR Status Assessment

HER2 and HR statuses were determined by IHC on tissue samples obtained from either primary tumors or metastatic sites. The HER2 status was classified as HER2-zero (IHC 0) or HER2-low (IHC 1+ or 2+ without HER2 gene amplification as confirmed by ISH). HER2 amplification was defined according to ASCO/CAP guidelines as a HER2/CEP17 ratio of ≥ 2.0 or an average HER2 gene copy number of ≥ 6 signals per cell. All IHC and ISH assessments were performed in accredited pathology laboratories at each center, following the ASCO/CAP criteria valid at the time of testing [4].

### 2.4. Outcome Definitions

PFS was defined as the time from the initiation of CDK4/6i treatment to radiological progression or death from any cause. OS was defined as the time from the initiation of CDK4/6i treatment to death from any cause. ORR was determined as the proportion of patients achieving a partial or complete response, while DCR was defined as the proportion of patients achieving stable disease, partial response, or complete response.

### 2.5. Statistical Analysis

All statistical analyses were performed using IBM SPSS Statistics Version 27.0 (Armonk, NY, USA). Categorical variables were expressed as frequencies and percentages, while continuous variables were reported as medians with interquartile ranges (IQRs) or ranges. Group comparisons were made using Pearson's Chi-squared or Fisher's exact test, as appropriate. Kaplan−Meier survival curves were generated for PFS and compared using the log-rank test.

Cox proportional hazards regression was used to calculate unadjusted hazard ratios (HRs) with 95% confidence intervals (CIs). Variables with *p* < 0.05 in univariate analysis were included in multivariate models to adjust for confounders. A two-sided *p* value < 0.05 was considered statistically significant.

## 3. Results

### 3.1. General Patient Characteristics

A total of 212 patients were included, with 47.7% (*n* = 100) classified as HER2-zero and 52.3% (*n* = 112) as HER2-low. The median age was 60 years (range: 24–85 years), with 34% aged ≥ 65 years. Most patients (71.2%) had an ECOG performance status (PS) of 0%, and 30.2% were premenopausal. De novo metastases were present in 48.1% (*n* = 102) of patients, and 51.9% (*n* = 110) had recurrent disease.

Visceral metastases were observed in 51.4% (*n* = 109), and 65.1% had two or more metastatic sites. Baseline characteristics were comparable between the HER2-zero and HER2-low groups. Ribociclib was administered to 57.9% of the patients. CDK4/6 were given as first-line therapy in 59.9% and as second-line or later therapy in 40.1%. ET consisted of aromatase inhibitors in 62.7% of the patients and fulvestrant in 37.3%, with a statistically significant difference between groups (*p*=0.016). Detailed clinical and pathological characteristics are summarized in Table 1.

### 3.2. PFS Comparisons

**Figure 1:**
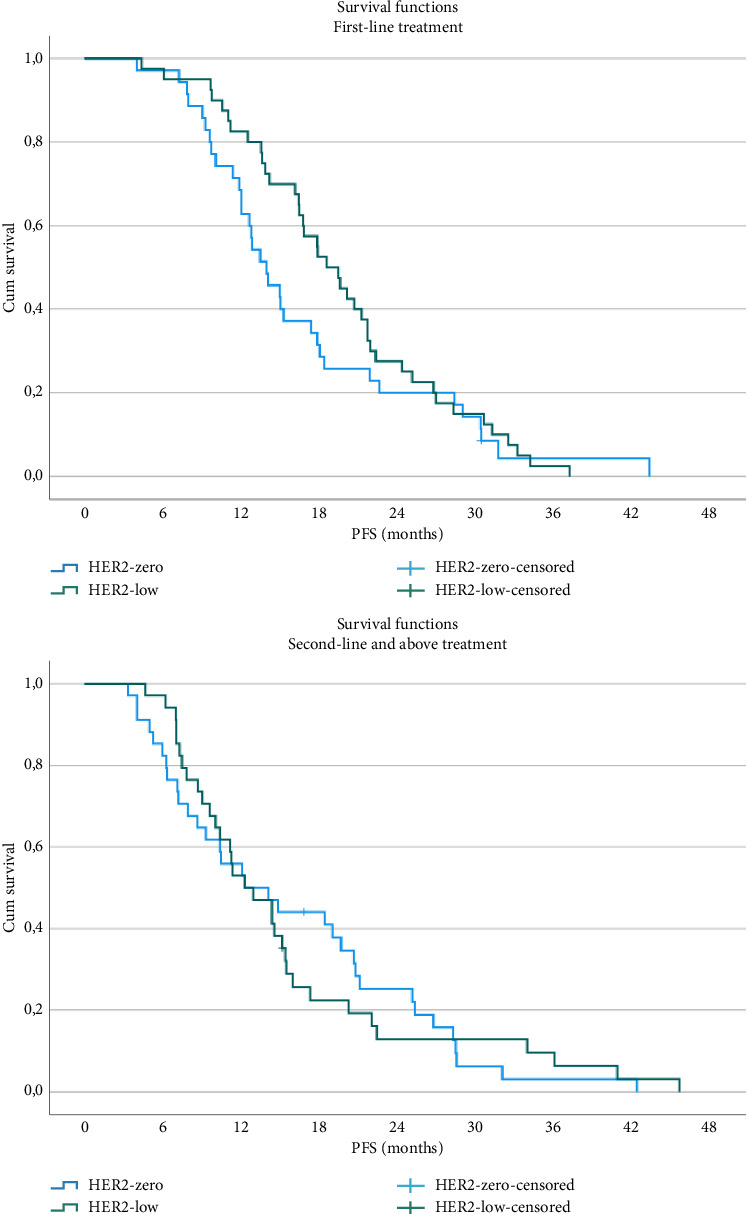
PFS by treatment line.

The median follow-up was 24.7 months. Overall, 106 patients (49.5%) experienced disease progression, with a median PFS of 15.0 months. The median PFS was 16.0 months in the HER2-low group and 13.9 months in the HER2-zero group, with no statistically significant difference (*p*=0.40). Among patients receiving CDK4/6i as first-line therapy, PFS was numerically longer in the HER2-low group compared with the HER2-zero group (18.6 months vs. 14.9 months; *p*=0.26) (Figure 1). For patients receiving second-line or later therapy, median PFS was identical between the two groups (12.3 months; *p*=0.98) (Figure 1). OS data were immature at the time of analysis, with 24.6% (*n* = 52) of the patients deceased.

### 3.3. Subgroup Analyses Within HER2-Low and HER2-Zero Groups

**Figure 2:**
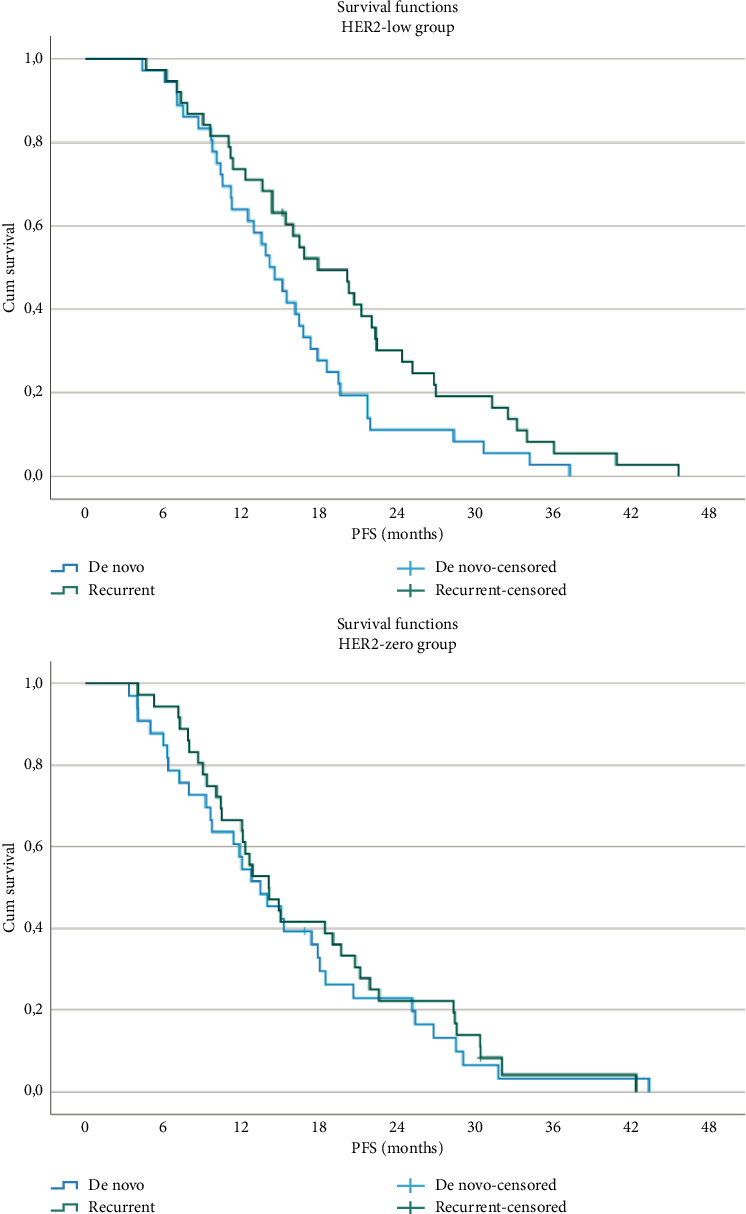
PFS by the HER2 status.

No significant differences in PFS were observed between the HER2-low and HER2-zero groups based on metastasis type (de novo vs. recurrent) (Figure 2). However, within the HER2-low group, patients with recurrent metastases had a significantly longer PFS compared with those with de novo metastases (17.9 months vs. 13.4 months; *p*=0.04) (Figure 2). No similar effect of metastasis type on PFS was noted in the HER2-zero group.

Among HER2-low patients, those with a single metastatic site had a significantly longer PFS than those with two or more metastatic sites (20.2 months vs. 14.1 months; *p*=0.02). In contrast, the number of metastatic sites did not impact PFS in the HER2-zero group. Detailed subgroup analysis results are presented in Table 2.

### 3.4. Objective Response and Disease Control Rates

The ORR was 62.0% in the HER2-zero group and 71.4% in the HER2-low group. The DCR was 82.0% and 86.6%, respectively. These differences were not statistically significant. OS data remained immature due to the low number of deaths (24.6%; *n* = 52) at the time of analysis.

### 3.5. HER2 IHC Subgroup Analysis

Patients were categorized into three subgroups based on HER2 IHC scores: HER2-zero, HER2-low 1+, and HER2-low 2+. The median PFS for these subgroups was 13.9 months, 14.4 months, and 16.4 months, respectively, with no statistically significant differences observed (*p*=0.55).

### 3.6. Prognostic Factors Influencing PFS

Univariate analysis identified the presence of visceral metastases, two or more metastatic sites, and the line of CDK4/6i therapy as candidate factors associated with PFS. In multivariate Cox regression analysis, the presence of visceral metastases (HR: 1.78; 95% CI: 1.23–2.58; and *p*=0.003) and the line of CDK4/6i therapy (HR: 1.43; 95% CI: 1.04–1.96; and *p*=0.029) remained independent prognostic factors for PFS. These findings are summarized in Table 3.

## 4. Discussion

In HR-positive/HER2-negative MBC, the combination of AI or fulvestrant with CDK4/6i is the current standard first-line treatment according to updated clinical guidelines [19]. In recent years, tumors scoring 1+ or 2+ by IHC but lacking gene amplification by ISH have been defined as the HER2-low subgroup within the broader HER2-negative category. This new classification has gained significance, particularly after the observed clinical activity of ADC therapies such as trastuzumab deruxtecan [6].

However, the biological differences between HER2-low and HER2-zero (IHC 0) tumors and their potential impact on response to standard treatments—particularly CDK4/6i—remain unclear. Therefore, our study aimed to compare PFS under combined CDK4/6i and ET between the HER2-low and HER2-zero subgroups.

Although treatment response according to HER2 expression level has not been clearly established in the literature, most studies have reported a slight trend toward longer PFS in HER2-zero patients [12, 20–27]. However, the majority of these differences have not reached statistical significance [23–27]. A meta-analysis including twelve studies demonstrated a significant advantage for HER2-zero patients receiving first-line therapy, with a pooled HR of 0.83 (95% CI: 0.74–0.94 and *p*=0.002). In contrast, no significant difference was observed between the groups in later-line treatments (HR: 0.92; 95% CI: 0.83–1.01; and *p*=0.08) [28]. Notably, the trend favoring HER2-zero in first-line settings reported in the meta-analysis contrasts with the opposite tendency observed in our study.

In our cohort, median PFS was longer in HER2-low patients compared with those with HER2-zero tumors (16.0 vs. 13.9 months; *p*=0.40), with a modest numerical difference observed in the first-line treatment subgroup (18.6 vs. 14.9 months; *p*=0.26). However, these differences did not reach statistical significance and should be interpreted as nonsignificant numerical variations. No difference was observed between the groups in later-line treatments (12.3 months; *p*=0.98). This discrepancy may be attributed to variations in patient populations, differences in ET regimens, or a higher prevalence of endocrine resistance in the HER2-zero group.

It is widely accepted in the literature that patients with de novo metastatic disease generally have a more favorable prognosis. This is thought to be related to their lack of prior exposure to systemic therapy and preserved endocrine sensitivity [29]. However, in our study, among patients in the HER2-low subgroup, those with recurrent metastatic disease had a longer median PFS compared with those with de novo metastases (17.9 vs. 13.4 months) although the difference did not reach statistical significance (*p*=0.26). Similarly, no significant difference was observed in the HER2-zero group (*p*=0.84). Although these differences were numerical, they should be interpreted with caution due to the limited sample size and possible confounders. The observed trend, despite differing from previous literature, lacks sufficient statistical power to suggest a meaningful conclusion.

In our overall analysis, both the number of metastatic sites and the presence of visceral metastases were found to have a significant prognostic impact on PFS. Limited metastatic burden and absence of visceral involvement were associated with longer PFS. This relationship was particularly evident in the HER2-low subgroup, where patients with only a single metastatic site had a significantly longer median PFS compared with those with multiple sites (20.2 vs. 14.1 months; *p*=0.02). In contrast, no such association was observed in the HER2-zero group. These findings are consistent with previous reports linking lower disease burden and absence of visceral metastases to better prognosis [12, 20–23, 26, 30, 31]. However, based on the current data, the difference in metastatic site number alone is unlikely to justify changes in treatment decisions. Nevertheless, the significant association observed in HER2-low patients with limited disease may point toward the existence of a biologically less aggressive tumor subset within this group though further biological validation is needed.

Although the objective response rate was higher in the HER2-low group (71.6% vs. 62%), this difference was numerical only and did not reach statistical significance (*p*=0.80). The literature presents conflicting evidence on this topic, with studies supporting either the HER2-zero or HER2-low group [20, 24, 26]. However, despite this inconsistency, a meta-analysis that accounted for methodological heterogeneity found no significant difference between the groups [28], aligning with our findings. Similarly, while some reports have suggested longer overall survival in HER2-zero patients, pooled analyses have not confirmed this. In our study, OS data remain immature, precluding interpretation at this stage. Taken together, these findings suggest that HER2 expression level alone is not a strong independent predictor of response to CDK4/6i-based therapies.

Molecular-level studies suggest that HER2-low tumors may possess a biologically distinct genetic background compared with HER2-zero tumors. The higher prevalence of ERBB2 alterations reported in this group indicates that HER2 expression may differ not only at the IHC level but also at the genomic level [32–34]. In addition, biological alterations such as PIK3CA mutations and FGFR1 amplifications have been observed more frequently in HER2-low tumors, supporting the notion that this subgroup may exhibit greater endocrine resistance and potentially worse prognosis [35–37]. However, the clinical impact of these molecular differences on treatment response and survival remains controversial [33]. In our study, HER2-low patients demonstrated longer PFS, which does not align with these biological expectations. This discrepancy suggests that HER2-low tumors may not represent a single, uniform biological entity and that unrecognized subpatterns might exist within this subgroup.

In addition, a bidirectional interaction between HER2 and HR signaling pathways has been proposed as another biological mechanism that may influence treatment response [15, 16]. This crosstalk can modulate HER2 protein expression independently of gene amplification and may thereby alter sensitivity to CDK4/6i. Preliminary evidence suggests that such signaling interactions in HER2-low tumors could result in distinct patterns of PFS. However, the clinical significance of this mechanism remains uncertain [8–14]. The absence of a clear impact of HER2 expression level on treatment response in our study suggests that neither genetic alterations nor signaling interactions alone may fully explain the behavior of HER2-low tumors and that more complex biological dynamics may be at play in this heterogeneous subgroup.

### 4.1. Study Limitations

The multicenter design of this study and the comparable number of patients in the HER2-low and HER2-zero groups ensured methodological balance in comparative analyses. However, the IHC-based classification of HER2 expression is subject to interobserver variability and potential discrepancies across centers, which may affect classification accuracy, despite the use of ASCO/CAP guidelines in accredited pathology laboratories. Future multicenter studies may benefit from centralized pathology review or digital pathology platforms to minimize such variability. The retrospective design carries inherent risks of selection bias and data inconsistency although these were partly mitigated by predefined inclusion criteria and the exclusion of incomplete records. The relatively small sample size and limited follow-up period further reduced statistical power, especially in subgroup analyses. The lack of molecular data (e.g., PIK3CA mutation) restricted the ability to explore biological differences in greater depth, particularly in relation to treatment resistance and tumor biology. In addition, the exclusion of agents not yet approved in our country, such as abemaciclib, limited treatment diversity. Furthermore, factors such as metastatic burden and visceral involvement—which independently influence treatment response—should be considered when interpreting outcomes.

## 5. Conclusion

Taken together, these findings suggest that HER2 expression level alone is not a definitive predictor of treatment response in HR-positive/HER2-negative MBC patients receiving CDK4/6i. While our results may not fully align with certain biological assumptions in the current literature, they reinforce the heterogeneous nature of the HER2-low subgroup and underscore the need for more in-depth molecular characterization. Relying solely on IHC scoring to define HER2-low status may be insufficient; more refined biological profiling could enhance therapeutic decision-making. Accordingly, the identification of advanced biomarkers for subclassifying HER2-negative patients should be considered a key objective for future research. Future prospective studies incorporating molecular profiling—particularly involving ERBB2, PIK3CA, and ESR1 alterations—are warranted to further elucidate the biological behavior and therapeutic implications of HER2-low tumors.

## Figures and Tables

**Table 1:** Comparison of baseline clinical and demographic characteristics between HER2-zero and HER2-low groups.

Parameters	HER2-zero (*n* = 100), *n* (%)	HER2-low (*n* = 112), *n* (%)	*p* value
Age, years, median (range)	59 (24–85)	60 (25–85)	0.30

*Age group*			0.15
< 65 years	61 (61%)	79 (70.5%)	
> 65 years	39 (39%)	33 (29.5%)	

*ECOG PS*			0.13
0	66 (66%)	83 (74.1%)	
≥ 1	34 (34%)	29 (25.9%)	

*Menopause status*			0.88
Premenopausal	31 (31%)	33 (25.9%)	
Postmenopausal	69 (69%)	79 (74.1%)	

*Disease status*			0.48
De novo metastatic	49 (49%)	53 (47.3%)	
Recurrent metastatic	51 (51%)	59 (52.7%)	

*Site of metastasis*			0.21
Visceral	56 (56%)	53 (47.3%)	
Nonvisceral	44 (44%)	59 (52.7%)	

*Number of metastatic site*			0.61
1 site	33 (33%)	41 (36.6%)	
≥ 2 sites	67 (67%)	71 (63.4%)	

*CDK4/6i*			0.34
Ribociclib	60 (60%)	63 (56.3%)	
Palbociclib	40 (40%)	49 (43.7%)	

*Line of treatment*			0.55
First line	60 (60%)	67 (59.8%)	
Second line or later	40 (40%)	45 (40.2%)	

*Treatment line*			0.64
First line	60 (60%)	67 (59.8%)	
Second line	32 (32%)	32 (28.6%)	
Third line or beyond	8 (8.0%)	13 (11.6%)	

*Endocrine therapy*			0.016
AI	54 (54%)	79 (70.5%)	
Fulvestran	46 (46%)	33 (29.5%)	

*Note:* Group comparisons were performed using Pearson's Chi-squared test for categorical variables and the Mann–Whitney *U* test for age. A *p* value < 0.05 was considered statistically significant.

Abbreviations: AI, aromatase inhibitor; CDK4/6i, cyclin-dependent kinase 4/6 inhibitor; ECOG PS, eastern cooperative oncology group performance status.

**Table 2:** PFS, ORR, and DCR analysis results.

Parameters	HER2-zero (*n* = 100)	HER2-low (*n* = 112)	*p* value
ORR	62%	71.6%	0.82

DCR	82%	86.6%	0.90

PFS (all group) (months)	13.9 (95% CI: 11.3–16.5)	16.0 (95% CI: 13.6–18.3)	0.40

PFS (months)			
1st line treatment	14.9 (95% CI: 11.8–17.2)	18.6 (95% CI: 15.9–21.3)	0.26
2nd line and above	12.3 (95% CI: 6–18.5)	12.3 (95% CI: 8.5–15.9)	0.98

PFS (months)			
De novo metastatic	13.7 (95% CI: 11.1–16.4)	13.4 (95% CI: 11.1–16.0)	0.87
Recurrent metastatic	14.2 (95% CI: 11.9–16.7)	17.9 (95% CI: 14.6–20.9)	0.24

PFS (months)			
1 metastatic site	14.5 (95% CI: 8.4–20.2)	20.2 (95% CI: 15.5–25)	0.11
2 and above met site	12.6 (95% CI: 9.1–16.2)	14.1 (95% CI: 9.9–18.4)	0.56

PFS (months)			
Use of ribociclib	14.1 (95% CI: 11.3–16.8)	17.3 (95% CI: 14.4–20.1)	0.61
Use of palbociclib	12.6 (95% CI: 9–16.8)	14.4 (95% CI: 10.2–18.6)	0.27

Abbreviations: DCR, disease control rate; ORR, objective response rate; PFS, progression-free survival.

**Table 3:** Prognostic factors for PFS in univariate and multivariate analysis.

Parameters	Univariate	*p* value	Multivariate	*p* value
	HR (95% CI)		HR (95% CI)	
Age < 65 vs. ≥ 65	0.95 (0.66–1.35)	0.766		

ECOG PS 0 vs. ≥ 1	1.43 (0.99–2.04)	0.052		

Menopause pre vs. post	1.32 (0.92–1.88)	0.131		

HER2-zero vs. HER2-low	0.89 (0.62–1.21)	0.4		

De novo vs. recurrent disease	0.73 (0.53–1.03)	0.07		

Nonvisceral vs. visceral metastasis	1.73 (1.23–2.44)	0.002	1.51 (1.05–2.18)	0.026

Number of metastatic site 1 vs. ≥ 2	1.52 (1.07–2.16)	0.02		

Number of metastatic site 1 vs. 2 vs. ≥ 3	Ref. 1.38 (0.94–2.20) 1.94 (1.22–3.08)	0.019 0.096 0.005	Ref. 1.21 (0.81–1.82) 1.66 (1.02–2.69)	0.124 0.348 0.041

Ribociclib vs. palbociclib	1.33 (0.95–1.86)	0.101		

First-line vs. subsequent lines treatment	1.22 (0.87–1.71)	0.246		

CDKI treatment in 1st line CDKI treatment in 2nd line CDKI treatment in 3rd line	Ref. 1.01 (0.70–1.46) 2.60 (1.54–4.41)	0.964 < 0.001	Ref. 0.48 (0.30–0.92 0.55 (0.30–1.03)	0.181 0.049 0.086

AI vs. fulvestran	1.30 (0.92–1.83)	0.132		

Abbreviations: AI, aromatase inhibitor; CDKI, cyclin-dependent kinase 4/6 inhibitor; ECOG PS, eastern cooperative oncology group performance status.

## Data Availability

The data that support the findings of this study are available on request from the corresponding author. The data are not publicly available due to privacy or ethical restrictions.
